# A novel chemopreventive mechanism of selenomethionine: Enhancement of APE1 enzyme activity via a Gadd45a, PCNA and APE1 protein complex that regulates p53-mediated base excision repair

**DOI:** 10.3892/or.2013.2613

**Published:** 2013-07-11

**Authors:** HWA JIN JUNG, HYE LIM KIM, YEO JIN KIM, JONG-IL WEON, YOUNG ROK SEO

**Affiliations:** 1 Department of Genetics, Albert Einstein College of Medicine, Bronx, NY 10461, USA; 2 Department of Life Science, Institute of Environmental Medicine for Green Chemistry, Dongguk University, Jung-gu, Seoul 100-715, Republic of Korea; 3 Department of Safety Engineering, Dongguk University, Gyeongju, Gyeongbuk 780-714, Republic of Korea

**Keywords:** selenomethionine, base excision repair, growth arrest and DNA damage-inducible protein 45A, proliferating cell nuclear antigen, APE1/Ref-1

## Abstract

Organic selenium compounds have been documented to play a role in cancer prevention. Our previous study showed that selenomethionine (SeMet) induces p53 activation without genotoxic effects including apoptosis and cell cycle arrest. In this study, we investigated the mechanism by which organic selenium compounds promote p53-mediated base excision repair (BER) activity. Our data demonstrated for the first time that the interaction between growth arrest and DNA damage-inducible protein 45A (Gadd45a), which is a p53-activated downstream gene, and two BER-mediated repair proteins, proliferating cell nuclear antigen (PCNA) and apurinic/apyrimidinic endonuclease (APE1/Ref-1), was significantly increased in a p53-dependent manner following treatment with organic selenium compounds. Furthermore, we observed that the activity of APE1 was significantly increased in a p53-dependent manner in response to the organic selenium compounds. These results suggest that BER activity is dependent on wild-type p53 activity and is mediated by the modulation of protein interactions between Gadd45a and repair proteins in response to organic selenium compounds. We propose that p53-dependent BER activity is a distinct chemopreventive mechanism mediated by organic selenium compounds, and that this may provide insight into the development of effective chemopreventive strategies against various oxidative stresses that contribute to a variety of human diseases, particularly cancer.

## Introduction

The cancer chemopreventive properties of selenium have been studied for over 20 years, and most studies have used rodent models of mammary carcinogenesis (1). The anticancer activity of both the inorganic and organic forms of selenium compounds has been studied. The prototype form, sodium selenite, exhibited a major drawback when its chemopreventive activity was tested; it is rapidly converted to hydrogen selenide, which generates DNA strand breaks and subsequent cytotoxicity (2–4). Among the organic forms of selenium, selenomethionine (SeMet) has been reported to have anticancer effects (2–6) and oral administration to humans as an anticancer drug has been approved. The efficacy of selenium, vitamin E, and β-carotene alone and in combination in the prevention of prostate cancer is currently being assessed in population-based clinical trials (7,8). These studies have been based on secondary analyses of large-scale chemoprevention trials for other cancers (9,10).

Recently, the chemopreventive mechanism of SeMet has been investigated via *in vitro* studies. Organic selenium SeMet compounds have been reported to activate tumor suppressor p53, and this activation may be one of the alternative chemopreventive mechanisms mediated by organic selenium (11). The protein p53 has been frequently referred to as the gatekeeper of the genome; it plays a well-established role in maintaining the stability of the genome by inducing either cell cycle arrest or apoptosis (12–14). The expression of p53 is induced by cellular stresses that cause DNA damage, and this increase in p53 expression promotes either cell cycle arrest or DNA repair (15). If the damage is too severe, apoptosis is induced (16). It has been established that p53 increases global DNA repair but not transcription-coupled nucleotide excision repair (NER) (17). Our previous study suggested that activation of p53 by SeMet plays a role in protecting cells against DNA damage induced by ultraviolet (UV) irradiation (11). Furthermore, our more recent study suggested that p53 and its downstream gene *gadd45a* are activated in cells following treatment with organic selenium, and this response may participate in restoring apurinic/apyrimidinic endonuclease (AP) sites during methyl methanesulfonate (MMS)-induced base excision repair (BER) (18).

Gadd45 (or Gadd45a, growth arrest and DNA damage-inducible gene) and p21 (Waf1/Cip1, a cyclin-dependent kinase inhibitor) are two well-established p53-regulated genes. Gadd45a binds to UV-damaged chromatin and affects the accessibility of sites of DNA damage to DNA repair machinery (19). An early study by Smith *et al*(20) showed that Gadd45a interacts with proliferating cell nuclear antigen (PCNA) and participates in NER; however, the precise mechanism and function of this interaction requires further characterization. A recent study suggested that PCNA interacts with APE1/Ref1, a key component of the BER pathway, in the nucleus (21). Taken together, these data suggest that Gadd45a affects BER activity via its interaction with PCNA and APE1/Ref-1. The BER pathway corrects DNA damage generated by ionizing radiation, simple alkylating agents, and endogenous hydrolytic and oxidative processes. BER is initiated by a monofunctional glycosylase, followed by AP endonuclease (APE)-mediated strand cleavage 5′ to the apurinic/apyrimidinic (AP) site. p53 has been reported to enhance methyl methanesulfonate (MMS)-induced BER activity. Our previous study suggested that *gadd45a*, a gene downstream of p53, participates in the BER pathway by interacting with BER-related proteins, such as PCNA and APE1/Ref-1 (22).

In the present study, we provide initial evidence that the p53-dependent interaction of Gadd45a with repair proteins is involved in the activation of BER in response to the organic selenium compound SeMet. Our study identified a novel chemopreventive property of the antioxidant selenium.

## Materials and methods

### Cell culture and treatment

We used isogenic human colon cancer cell lines carrying wild-type p53 and mutant p53 derivatives, in which p53 function was abrogated by the introduction of a dominant-negative p53 mutant allele (codon 143; valine to alanine) (23). Cells were cultured in RPMI-1640 medium supplemented with 10% fetal bovine serum (FBS) (both from Gibco-BRL, Carlsbad, CA, USA) and antibiotics. RKO cells were treated with organic selenium (20 and 40 μM) for 16 h at 37°C and incubated in 5% CO_2_:95% air.

### Preparation of DNA substrates

Oligonucleotides were 17-mers containing either tetrahydrofuran (THF) or a normal nucleotide (dA) at position 9. Complementary oligos with a T opposite the THF were also used. The sequences were as follows: 5′-AGCATTCGXGACTGGGT-3′, in which the X indicates THF, and dA; 5′-ACCCAGTCTCGAATGCT-3′ was the complementary strand. Spacer CE phosphoramidite (a tetrahydrofuran derivative) was purchased from Integrated DNA Technologies (IDT) Inc. (USA). The oligonucleotides were radiolabeled at their 5′ end using [γ-^32^P]-ATP (Amersham Life Sciences, USA) and T4 polynucleotide kinase (Promega Corporation, Madison, WI, USA). The oligonucleotides were annealed by heating them at 90°C, followed by cooling to room temperature for several hours.

### Assay measuring AP endonuclease activity in cell lysates

Crude cell extracts were assayed for AP endonuclease activity using the double-stranded oligonucleotide as a substrate. Reactions were performed in 10 mM Tris-HCl at pH 7.5, 50 mM NaCl and 1 mM EDTA. The reaction mixtures containing 25 μg total protein and 5′ radiolabeled substrate (10^4^ cpm, ≈0.75 pmol) were incubated at 37°C for 30 min. The stop solution (98% formamide, 10 mM EDTA, 0.025% bromophenol blue and 0.025% xylene cyanol) was added to the reactions, followed by incubation on ice. The products were separated on 20% denaturing polyacrylamide gels, and autoradiography was carried out for 15 h at −70°C using Kodak X-Omat AR film (Eastman Kodak, Rochester, NY, USA).

### Transfection of p53 siRNA

siRNA duplexes targeting p53 were designed and synthesized by Dharmacon Inc. (Chicago, IL, USA). RKO cells were transfected using Oligofectamine reagent (Invitrogen GmbH, Karlsruhe, Germany), according to the manufacturer’s instructions, with single-strand sense and antisense RNA oligonucleotides (human p53 sense RNA, 5′-GAGGUUGGCUCUGACUGUAUU-3′ and antisense RNA, 5′-UACAGUCAGAGCCAACCUCUU-3′). For each well, 5 μl of 20 nmol of the oligonucleotides was diluted with 175 μl of serum-free RPMI-1640 medium. Then 4 μl of Oligofectamine (Invitrogen GmbH) was diluted and incubated for 10 min in 15 μl of serum-free RPMI-1640 medium. The 2 solutions were combined and incubated at room temperature for 15 min. This solution was incubated with RKO cells for 4 h, and then the medium was replaced with RPMI-1640 containing 10% FBS.

### APE1/Ref1 immunostaining

For APE1/Ref1 staining, cells were grown on coverslips and fixed with ice-cold 100% methanol for 30 min at −20°C. The cells were then dehydrated with ice-cold 100% acetone for 1 sec and washed 3 times with PBS. The fixed cells were incubated with an APE1/Ref-1 mouse antibody (clone Ab82) (NeoMarkers, Fremont, CA, USA) diluted 1:3,000 in BSA solution (0.5% bovine serum albumin in PBS) for 4 h at room temperature. After incubation with the primary antibody, the coverslips were washed 4 times for 5 min each with PBS containing 0.1% Tween-20. Then the cells were incubated with fluorescein isothiocyanate (FITC)-conjugated anti-mouse secondary antibody (Vector Laboratories, Burlingame, CA, USA) for 1 h at room temperature, followed by 6 5-min washes with shaking. Coverslips were mounted on standard microscope slides with mounting media containing DAPI (Vector Laboratories). Images were viewed and captured using a fluorescence microscope (Nikon Eclipse 50i and NIS-Elements F 2.20, Nikon, Japan).

### Immunoblotting and immunoprecipitation

All western blotting was conducted as previously described by Smith *et al*(24). The following antibodies were used: APE1/Ref1 (ab82) (NeoMarkers); PCNA (PC10); Gadd45a (Ab H165) (both from Santa Cruz Biotechnology, Inc., Santa Cruz, CA, USA) and glyceraldehyde 3-phosphate dehydrogenase (GAPDH) (LabFrontier, Seoul, Korea). The proteins were detected using horseradish peroxidase-conjugated secondary antibodies (Sigma-Aldrich, St. Louis, MO, USA), followed by enhanced chemiluminescence (Pierce Biotechnology, Inc., Rockford, IL, USA).

Cells were lysed in immunoprecipitation (IP) buffer [1% Nonidet P40, 0.1% SDS, 50 mM Tris-HCl (pH 7.8), 150 mM NaCl, 1 mM DTT and 0.5 mM EDTA containing protease inhibitors (Complete™; Roche Molecular Biochemicals, Mannheim, Germany)] for 30 min at 4°C. The samples were sonicated for 5 sec. Equal amounts of protein extracts were precleared by incubating them with 30 μl of protein A/G agarose beads (Santa Cruz Biotechnology, Inc.) for 1 h at 4°C with shaking. The precleared supernatants were aliquoted for IP with 1 μg of either rabbit anti-Gadd45a antibody, mouse anti-PCNA antibody, or rabbit anti-APE1/Ref-1 antibody for 4 h at 4°C. These antibody-containing lysates were incubated with 40 μl of protein A/G agarose beads (Santa Cruz Biotechnology, Inc.) overnight at 4°C. The beads were washed 4 times with IP buffer. The immunoprecipitated proteins were resuspended in 4X sample buffer, resolved by 10% sodium dodecyl sulfate-polyacrylamide gel electrophoresis (SDS-PAGE), and electrotransferred to membranes before western blotting with the indicated antibodies.

## Results

### Increased interaction of PCNA and APE1/Ref-1 with Gadd45a in response to SeMet

**Figure 1:**
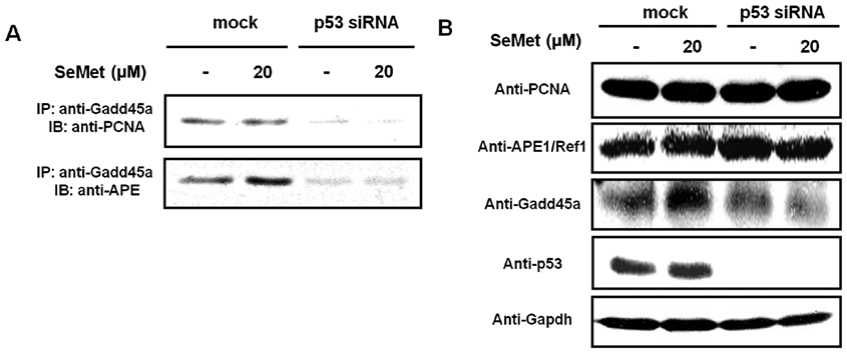
p53-dependent interaction of Gadd45a, APE1 and PCNA in response to SeMet. (A) Co-immunoprecipitation of Gadd45a, PCNA and APE1 from mock-treated RKO cells and p53 siRNA-treated RKO cells. Anti-Gadd45a antibody (Ab) was used for immunoprecipitation. An anti-PCNA or anti-APE1 primary Ab was used for western blotting. A secondary Ab conjugated to a near-infrared dye was used to detect the bands corresponding to PCNA or APE1. In the cells with reduced p53 expression, the interaction of PCNA and APE1 with Gadd45a was significantly decreased in response to SeMet. (B) APE1, PCNA and Gadd45a were detected using western blotting. GAPDH was used as a marker for equal protein loading.

We first examined whether an interaction exists between Gadd45a, APE1/Ref1 and PCNA following the treatment of SeMet (20 μM as a non-genotoxic dose in which apoptosis and cell cycle arrest are not induced, data not shown). We used the technique of immunoprecipitation from human colon cancer RKO cell lysates to evaluate this potential interaction. An interaction between Gadd45a and PCNA was previously demonstrated; therefore, we extended this study to determine whether Gadd45a also interacts with PCNA and APE1/Ref1 in the context of BER activation. Gadd45a strongly interacted with PCNA in mock-treated RKO cells in response to organic selenium (Fig. 1), whereas almost no interaction took place between Gadd45a and PCNA in p53 siRNA-treated RKO cells treated with organic selenium. In addition, the interaction between Gadd45a and APE1/Ref-1 was dramatically reduced in p53 siRNA-treated cells (Fig. 1A). The expression of Gadd45a, APE1/Ref-1 and PCNA in the whole cell lysates and siRNA-mediated knockdown of p53 were confirmed by western blotting (Fig. 1B). Our data suggest that the interaction between Gadd45a, PCNA and APE1/Ref-1 is promoted in a p53-dependent manner in response to organic selenium.

### Increased p53-dependent AP endonuclease activity in response to SeMet

**Figure 2:**
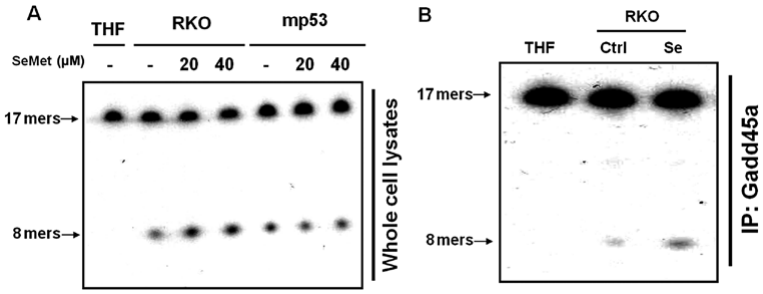
Activity of AP endonuclease in response to SeMet. (A) The AP endonuclease activity of APE1 on the THF-containing double stranded DNA substrate was assayed in RKO cells with wild-type and mutant p53. The AP endonuclease activity (the 8-mer represents the AP endonuclease cleavage product) was considerably higher in wild-type p53 cells treated with SeMet (20 and 40 μM), as compared to SeMet-treated mutant p53 cells. (B) The Gadd45a-immunoprecipitated complex was used in the APE assay. Cleavage of the THF-containing DNA substrate was increased in response to SeMet, indicating that the activity of APE1 was promoted by its SeMet-induced interaction with Gadd45a.

We examined cell extracts for the presence of enzymes that could act on damaged bases or their analogues. Tetrahydrofuran (THF)-containing oligonucleotide duplexes were used as substrates to assess AP endonuclease activity. In mammalian cells, APE1, the major AP endonuclease, is able to cleave DNA duplexes containing THF. The cleavage of 17-mer THF-containing duplex oligonucleotides into 8-mers by APE1 was dramatically increased in the SeMet-treated cells, as compared to untreated cells. These data suggest that the organic selenium-treated cells had greater APE1 activity than untreated cells (Fig. 2A). Furthermore, the cells that expressed mutant p53 did not exhibit any alteration in the level of APE1 activity in the presence of SeMet (Fig. 2A). In addition, we demonstrated that the interaction of Gadd45a with APE1, as assessed by immunoprecipitation, increased the activity of APE1 in response to SeMet (Fig. 2B). Taken together, these data suggest that the AP endonuclease activity required to remove the abasic site during BER increased as a result of its interaction with Gadd45a in response to the antioxidant organic selenium.

### APE1 localization is not altered in the presence of SeMet

**Figure 3:**
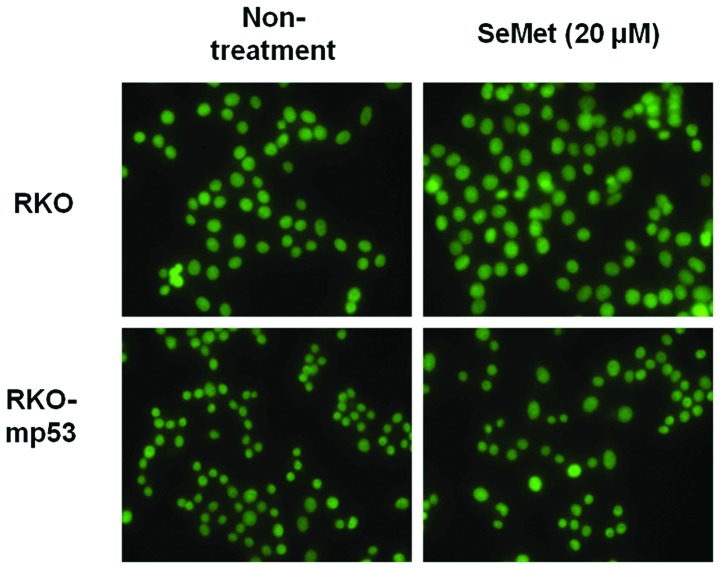
APE1 localization in RKO wild-type and mutant p53 cells in response to SeMet. Immunostaining for APE1 in wild-type and mutant p53 RKO cells treated with organic selenium. The localization of APE1 in RKO cells was not altered by lack of p53 function. The APE1 staining images were captured using a confocal microscope (x400 magnifications, Carl Zeiss, Jena, Germany).

APE1/Ref-1 is recruited to sites of DNA damage and becomes associated with nuclear substructures. We examined whether the localization of APE1/Ref-1 is regulated by p53 in response to SeMet using an isogenic pair of RKO cell lines with wild-type and mutant p53. APE1/Ref-1 immunostaining showed that APE1/Ref-1 was localized to the nucleus in both the wild-type and p53 mutant RKO cell lines irrespective of organic selenium treatment (Fig. 3). These data suggest that altered localization of APE1 is not involved in the p53-mediated promotion of BER activity in response to organic selenium.

## Discussion

BER activity has been reported to increase when DNA damage is induced by ionizing radiation and simple alkylating agents, as well as free radicals generated from endogenous hydrolytic and oxidative processes (25). BER converts diverse base lesions into common intermediate apurinic/apyrimidinic (AP) sites. These AP sites have been reported to spontaneously arise at a substantial rate and are thought to be one of the most frequent types of DNA lesions (26). It is estimated that the spontaneous hydrolytic loss of purines generates ~10,000 AP sites per day in mammalian cells (27,28). Therefore, the BER pathway is critical for handling the diverse lesions produced as a result of the intrinsic instability of DNA or by the insults of various endogenous and exogenous reactive oxygen species. Defects in the BER process are associated with an increased susceptibility to cancer and neurodegenerative disorders.

Although the SELECT study, one of the largest human cancer prevention clinical trials, did not find evidence that SeMet prevents prostate cancer (29), increasing evidence indicates that other micronutrient combinations may have beneficial effects. Recently, Qiao *et al*(7) suggested that the beneficial effects of Se, vitamin E, and β-carotene on total and gastric cancer mortality were still evident up to 10 years after supplementation and were consistently greater in participants younger than 55. This general population nutrition intervention trial, conducted in Linxian, China, where nutritional deficiencies and high cancer rates are common, suggest that the basal level of minerals including Se is critical to cancer incidence.

The interaction of BER repair proteins has been investigated in the context of organic selenium-induced activation of the BER pathway. Most reports have proposed a direct role for p53 in this process via its interaction with BER-associated proteins, including DNA polymerase β and AP endonuclease (30–33). In contrast, p53 controls NER by transcriptionally regulating NER proteins, such as p48XPE and XPC (34). Indeed, we previously demonstrated that Gadd45a-null cells exhibit a defect in activating the BER pathway, which is comparable to that previously observed in p53-null cells. Since isogenic cell lines were used for these studies, these data suggest that Gadd45a could play a major role in p53-dependent activation of BER (22). Based on these observations, we proposed that Gadd45a participates in the BER pathway by interacting with BER pathway proteins, including PCNA and APE1/Ref1 (22). In fact, Gadd45a was reported to interact with PCNA and increased NER in response to UV irradiation (20,35). The data in Fig. 1A and B indicate that the interaction of Gadd45a with PCNA and APE1/Ref-1 was significantly increased in p53 wild-type cells treated with organic selenium but not in p53 siRNA-treated cells. These data suggest that organic selenium modulates the activity of BER via the promotion of an interaction between p53 downstream genes Gadd45a and PCNA with APE1/Ref-1. However, the mechanism(s) by which p53 activity affects Gadd45a expression in organic selenium-treated cells has not yet been clarified.

Recently, our group suggested that the antioxidant organic selenium recovers the base damage induced by the alkylating agent MMS in p53 wild-type cells, as well as the nucleotide damage induced by UV irradiation (11,36). In a previous study in which we measured the fraction of unrepaired AP sites, these pathways were restored more extensively in wild-type p53 cells that were pretreated with organic selenium than in untreated cells (18). Treatment of cells with the alkylating agent MMS primarily induces N7-methylguanine and 3-methyladenine expression during the BER process. Then N-methylpurine DNA glycosylase expression is induced, which generates apurinic sites that are recognized by APE1. APE1 incises the damaged strand immediately at 5′ to the AP site. Here, we determined that the APE1 enzyme activity was considerably greater in p53 wild-type cells that were treated with organic selenium than in mutant p53 cells (Fig. 2A). These data suggest that basal BER activity may be promoted via the p53-dependent activation of APE1. In addition, the increased activity induced by SeMet was associated with Gadd45a-specific binding to APE1 (Fig. 2B). Indeed, APE1/Ref-1 has been reported to mediate the cysteine reduction of a number of transcription factors, including c-jun and p53 (37). Furthermore, APE1 activity induced by the other form of organic selenium, methylseleninic acid (MSeA), which has shown chemopreventive properties, was markedly increased in Gadd45a-specific captured cells (data not shown). These results suggest the existence of a feedback mechanism whereby organic selenium-mediated activation of p53 can modulate APE1/Ref-1 localization. However, the localization of the APE1 protein was not altered in the p53 siRNA-treated cells (Fig. 3), suggesting that the activity of the APE1 enzyme may be increased via processes other than protein shuttling. Our previous study reported that nuclear APE1 in gadd45a-knockdown cells was released into the cytosol, and therefore, the mechanism responsible for p53-dependent modulation of APE activity requires further exploration (22).

**Figure 4:**
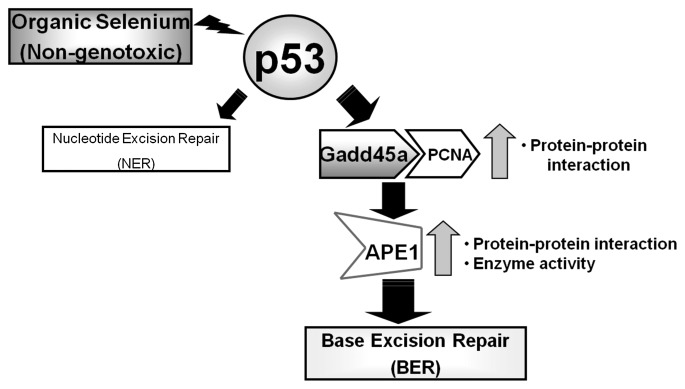
Model for the role of p53-dependent Gadd45a and APE1 interaction in response to organic selenium. In a previous study, p53-mediated BER activity was shown to be increased in response to organic selenium (22). In this study, we suggest an alternative chemopreventive mechanism of organic selenium, in which the p53 downstream gene *gadd45a* is activated by organic selenium, and this promotes the activation of BER via its increased interaction with PCNA and APE1.

Based on the results of this study, we propose that the promotion of BER activity by a p53-dependent pathway is mediated by an interaction between Gadd45a, PCNA and APE1 in the presence of organic selenium that results in increased APE1 activity (Fig. 4). This study, in combination with our previous study, which demonstrated that organic selenium promoted UV-induced NER (11), supports the rationale for targeting p53-mediated DNA repair as a means of cancer prevention. Our study supports the possibility of a novel chemopreventive mechanism of organic selenium in preventing mutagenesis induced by various oxidative metabolites that cause detrimental DNA base changes in normal cells.
